# The selection of COVID-19 epidemic prevention and control programs based on group decision-making

**DOI:** 10.1007/s40747-021-00620-6

**Published:** 2022-01-04

**Authors:** Chunsheng Cui, Baiqiu Li, Liu Wang

**Affiliations:** College of Computer and Information Engineering, Henan University of Economics and Law, 180 Jinshui East Road, Zhengzhou, 450046 Henan China

**Keywords:** Epidemic prevention and control, Group decision making, Alternative ranking, Consensus reaching

## Abstract

COVID-19 has been wreaking havoc on the world for close to two years. As the virus continues to mutate, epidemic prevention and control has become a long and experienced war. In the face of the sudden spread of virus strains, how to quickly and effectively formulate prevention and control plans are essential to ensuring the safety and social stability of cities. This paper is based on the characteristics, namely, its persistence and the high transmissibility of mutated strains, as well as the database of epidemic prevention and control plans formed as part of the existing prevention and control measures. Then, epidemic prevention experts select effective alternatives from the program database and rank their preferences through the preliminary analysis of the local epidemic situation. The process of the integration scheme aims to minimize the differences in an effort to maximize the needs of the local epidemic. Once the consensus ranking of the scheme is obtained, the final prevention and control scheme can be determined. The proposed method of this paper can optimize the opinions of the epidemic prevention expert group and form a consensus decision, whilst also saving time by carrying out the work effectively, which is of certain practical significance to the prevention and control effect of local outbreaks.

## Introduction

At the end of 2019, the COVID-19 virus was first observed in Wuhan, China, later spreading to many countries and regions around the world [16]. At present, the novel coronavirus has produced many mutated strains that posed a serious threat to health safety globally, such as the Alpha, Beta, Gamma, Delta, and Lambda variants. The transmission rate and carrying capacity of the mutated virus far exceed the original virus, which frustrates epidemic prevention and control attempts. Based on the ongoing international epidemic situation, human beings look set to coexist with the novel coronavirus for a long time. As such, the work of epidemic prevention and control is becoming the norm. In May 2021, Guangzhou became the first city in China to fight Delta variant strains and effectively controlled the outbreak in the space of a month. Now the Delta variant strains are spreading rapidly in China and even the world, posing new challenges for mankind to overcome the epidemic. Epidemic prevention and control experts are studying gene sequences, antibodies, prediction models and other aspects in the fight against the COVID-19 virus. For example, the United States used artificial intelligence technology to screen out dozens of drugs that can deal with the new coronavirus [17]. The UK was the first to approve oral anti-coronavirus drugs. Also, Castillo O and Melin P proposed a Forecast model and a hybrid intelligent fuzzy fractal method to accurately classify countries based on the complexity of COVID-19 time series data [3, 4]. Many other scholars put forward a prediction model for the spread of the new coronavirus, which has contributed to the prediction of the epidemic [1, 18, 21]. The speed of transmission was faster than previous outbreaks, which once again prompted epidemic prevention and control experts from all countries to reconsider their strategies. Based on this, this paper considers how to quickly and effectively formulate prevention and control solutions to ensure urban safety and social stability in light of the continually mutating novel coronavirus.

When tackling major issues, it is necessary to undertake group decision-making to determine the final solution. Group decision-making involves integrating the preferences of multiple decision-makers into group preferences [11, 12]. What’s more, it is considered an efficient and accurate means to quickly arrive at optimal decisions is to have many experts from various fields devote time and energy to studying group decision-making in-depth [24, 27]. The first group of experts on group decision-making focus on voting [19]. Typically, the simple majority principle is applied to make decisions as a group [2, 8]. Group decision-making is designed to achieve a consensus among the various opinions that are put forward. A consensus is both conducive to the smooth implementation of group programs and also conducive to building harmonious interpersonal relationships within the organization [22]. Next, experts studying group decision-making try to maximize consensus to rank alternatives. At this stage, many scholars measure the preferences of experts based on fuzzy quantitative analysis [9, 10, 20]. Due to the influence of objective factors such as the uncertainty of things themselves and the subjective factors such as the knowledge structure and judgment level of decision-makers, the views of decision-makers tend to be divergent [7]. Cook et al. [15]proposed the Borda-Kendall method to measure consensus for ranking alternatives, however, distance-based approaches sometimes fail to properly reflect consensus in group decision-making. Meanwhile, Huo et al. [23]put forward a premetric-based concept to express the various opinions of experts, identify the differences among these expert opinions when ranking the alternatives, negotiate and adjust the preferences of experts with the largest differences, and finally obtain the ranking of the alternatives with the smallest differences to make up for the deficiency of the distance-based identification method. Later, Hou’s subsequent paper followed a post-consensus analysis of the methodology to facilitate new insights into the alternatives [26]. Group decision-making has readily been applied in various areas including failure mode and impacts analysis, supply chain management, and water resources management, among others. More recently, group decision-making has also been studied in relation to emergency decision-making and disaster management [13, 25]. However, little research has been carried out to look into how to make decisions pertaining to prevention and control solutions in the face of the rapidly developing COVID-19 situation.

With the rapid development of mutated COVID-19 strains, the timely prevention and control of the new outbreaks is the focus of the current epidemic prevention and control efforts. As the new variants are spreading quicker than their antecedents, once the source of an infection in the outbreak is identified as a variant strain, the relevant government departments should quickly analyze the situation and organize experts to put forward prevention and control solutions to tackle the local epidemic. Against this backdrop, this paper studies how the expert group selects suitable alternatives for this epidemic according to the existing epidemic prevention schemes. Then, according to the method proposed by Huo et al. [6, 14, 23], the solution that the expert group considers most to meet the needs of the local epidemic should be selected. The contributions of this article are as follows: First of all, this paper proposes a preference-based group decision-making method, which includes experts negotiating and modifying links for the largest disputes, which not only speeds up the decision-making speed of experts on epidemic prevention and control, but also improves the application rate of the final prevention and control plan. Second, this article summarizes options for nucleic acid testing suitable for the current stage of the epidemic based on the existing experience in epidemic prevention and control. At the same time, this article takes the nucleic acid detection prevention and control program as an example to verify the applicability of the model and has practical significance for solving the current epidemic prevention and control. Therefore, this method is very suitable for decision-making in the ever-changing new crown epidemic.

The rest of the paper is arranged as follows: The second section introduces the group decision-making methods used in this paper, including the concepts of preference map, consensus gap, and consensus evaluation sequence, and so on. The third section introduces the general process of group decision-making of epidemic control programs from the perspectives of epidemic persistence and the rapid transmission of variant strains. The fourth section applies the previously outlined decision-making method to the selection of control schemes for the spread of mutant strains, taking nucleic acid testing (NAT) as an example. At the same time, compared with the existing group decision-making methods, the method used in this paper is better suited to decision-making related to epidemic prevention and control programs. Finally, the fifth section puts forward the conclusions and prospects of this paper.

## Theoretical basis

This paper studies how to rank the epidemic preventive measures based on expert preferences. This section briefly describes the basic theories and concepts used [13–15].

Let $E=\{e_1,e_2,\dots ,e_m\}$ be the set of the expert group and $A=\{a_1,a_2,\dots ,$ $a_n\}$ be the alternatives to be ranked, where $1<m<+\infty$ and $1<n<+\infty$. Assuming that expert preferences are considered to allow a parallel sequencing, and the alternatives in a tie are arranged in the same positions, which are continuous positive integers.

### Definition 1

[13]. A sequence $(S_i)_{n \times 1}$ is called the preference map (PM) of the alternation set *A* with respect to the order relation $\preceq$, if and only if the following is true: $S_i = \{|P_i|+1,|P_i|+2,\dots ,|P_i|+|Q_i|\}$, where $P_i=\{a_j|a_j\in A, a_j\succ a_i\}$ and $Q_i=\{a_k|a_k\in A, a_i\sim a_k\}$.

Definition 1 is based on the following two definitions:

A sequence $(P_i)_{n \times 1}$ is called the predominance sequence of the alternation set *A* with respect to the order relation $\preceq$, if and only if the following is true: $P_i=\{a_j|a_j\in A, a_j\succ a_i\}$.

A sequence $(Q_i)_{n \times 1}$ is called the indifference sequence of the alternation set *A* with respect to the order relation $\preceq$, if and only if the following is true: $Q_i=\{a_k|a_k\in A, a_i\sim a_k\}$.

### Definition 2

[13]. Assume that $V^{(1)} = (V^{(1)}_i)_{n \times 1}$, $V^{(2)} = (V^{(2)}_i)_{n \times 1}$ are two PMs of the experts, then the consensus gap between them is defined as follows:

$$\begin{aligned}&\varDelta (V^{(1)},V^{(2)}) = \sum _{i=0}^{n}\delta (V^{(1)},V^{(2)})\\&\quad =\!\sum _{i=1}^{n}\max \{0,\min V^{(1)}_i \!-\! \max V^{(2)}_i, \min V^{(2)}_i \!-\! \max V^{(1)}_i\}. \end{aligned}$$

The consensus gap index is a premetric, which only satisfies the properties of non-negativity and symmetry, so as to represent the disagreement between the two preference maps.

Moreover, a dispute matrix that is associated with the expert’s disagreements on alternatives is defined by $\mathrm{DispM} = (S_{ik})_{n \times n}$, where $\mathrm{DispM} = (S_{ik})_{n \times n}$ represents the total gap of the experts if $a_i$ is to be ranked at position k.

### Definition 3

[13, 14]. Assume that $V^{(1)} = (V^{(1)}_i)_{n \times 1}, V^{(2)} = (V^{(2)}_i)_{n \times 1}, \dots ,$ $V^{(m)} = (V^{(m)}_i)_{n \times 1}$ are the PMs of the experts. The experts are in consensus if, and only if $\forall i(V^{(1)}_i\cap V^{(2)}_i\cap \dots \cap V^{(m)}_i \ne \varnothing )$. The consensus ranking is $(W_i)$, where

$$\begin{aligned} \begin{pmatrix} W_1\\ W_2\\ \vdots \\ W_n \end{pmatrix}= & {} \begin{pmatrix} V^{(1)}_1\\ V^{(1)}_2\\ \vdots \\ V^{(1)}_n \end{pmatrix} \cap \begin{pmatrix} V^{(2)}_1\\ V^{(2)}_2\\ \vdots \\ V^{(2)}_n \end{pmatrix} \cap \dots \cap \begin{pmatrix} V^{(m)}_1\\ V^{(m)}_2\\ \vdots \\ V^{(m)}_n \end{pmatrix} \\= & {} \begin{pmatrix} \bigcap ^m_{k=1}V^{(k)}_1\\ \bigcap ^m_{k=1}V^{(k)}_2\\ \vdots \\ \bigcap ^m_{k=1}V^{(k)}_n \end{pmatrix}. \end{aligned}$$

The consensus gap between each pair of PMs represents the differences between the two experts, and the disagreement matrix represents the disagreement among all experts.

The disagreement matrix is defined as:

$$\begin{aligned} D=(\varDelta _{jk})_(m \times m), \quad \text {where } \varDelta _{jk}=\varDelta (V^{(j)},V^{(k)}). \end{aligned}$$

### Definition 4

[15]. The Consensus Evaluation Sequence (CES) is defined as follows:

$$\begin{aligned} \mathrm{CES}=[\mathrm{GCI}; \mathrm{MDP}, \mathrm{PDisaI}; \mathrm{MDA}, \mathrm{MDispI}]. \end{aligned}$$

The consensus evaluation sequence (CES) represents the degree of expert consensus on the ranking of alternatives. It contains the group consensus index (GCI), the maximum disagreement pairs (MDP), the pairwise disagreement index (PDisaI), the maximum dispute alternatives (MDA) and the maximum dispute index (MDisaI).

(1) GCI indicates the proportion of the number of expert pairs that reach consensus among all possible expert pairs.It is defined as follows:

1 $$\begin{aligned} \mathrm{GCI}=\frac{2\sum _{i=1}^{m-1}\sum _{j=j+1}^{m}\rho _{ij}}{m(m-1)},\text {where}\ \rho _{ij} \!=\! {\left\{ \begin{array}{ll} 1,&{}\varDelta _{ij} = 0,\\ 0,&{}\text {others}. \end{array}\right. }\nonumber \\ \end{aligned}$$

The range of values of the GCI is [0, 1]. $GCI=1$ expresses that the experts reach a complete consensus, and the bigger GCI expresses that the higher the consensus level of the experts.

(2) PDisaI indicates the biggest disagreement among the experts.It is defined as follows:

2 $$\begin{aligned} \mathrm{PDisaI} = \max \limits _j\{ \max \limits _k\{\varDelta _{jk}|j<k\}\}. \end{aligned}$$

$\mathrm{PDisaI}=0$ indicates that the experts have a complete consensus on each choice; otherwise, PDisaI represents the largest inconsistency value of the expert group.

(3) MDP implies the expert pairs with the biggest differences.It is defined as follows:

3 $$\begin{aligned} \mathrm{MDP} = \{(j,k)|\varDelta _{jk}=\mathrm{PDisaI}, j<k, \varDelta _{jk}<0\}. \end{aligned}$$

MDP represents the subscript-pair set of expert pairs with that index value, if PDisaI is not 0.

(4) MDispI indicates the most controversial index of experts on alternatives.It is defined as follows:

4 $$\begin{aligned} \mathrm{MDispI} = \max \limits _i\min \limits _k\{S_{ik}\}. \end{aligned}$$

$\mathrm{MDispI}=0$ shows that the experts have no controversy about the ranking of each alternative; otherwise, MDispI represents the maximum controversial value of the experts for the alternatives.

(5) MDA implies the alternative with the biggest disagreement.It is defined as follows:

5 $$\begin{aligned} \mathrm{MDA}=\{i|S_{ik} = \mathrm{MDispI}, S_{ik}>0\}. \end{aligned}$$

MDA represents the subscript set of alternatives with that index value, if MDisaI is not 0.

Decision-makers can identify whether the group of experts fully reach a consensus from the CES. Also, decision-makers can obtain expert pairs who have the maximum disagreement and the alternatives which have the maximum controversial value, if the group of experts is not in consensus.

## General process of group decision-making in epidemic prevention and control programs

At present, the ongoing epidemic is caused by the spread of new coronavirus. This virus is continually exhibiting new characteristics, such as its long duration, wide range, strong transmissibility, high viral load in infected patients, the rapid development of symptoms in patients, and so on. The epidemic prevention and control measures need to be formulated and implemented according to the changing situation of the epidemic. Therefore, the epidemic prevention and control programs must be quickly developed, in addition to being effective and enforceable. Since the novel coronavirus mutates rapidly, successfully controlling the epidemic relates to the immediate safety of society and the maintenance of social stability. When formulating epidemic prevention and control policies, the government ought to screen out the existing epidemic prevention and control schemes according to the local epidemic situation, rather than simply copying the solutions adopted at the last outbreak. Next, experts should be asked to make group decisions and choose the best alternative to meet local needs.

The goal of group decision-making is to comprehensively consider the opinions of various experts, integrate individual decision-making into group decision-making, and ultimately reach expert consensus to the maximum extent so as to determine the most feasible alternative. Group decision-making can adopt the opinions of experts from various aspects to transcend the limitations of individual knowledge and thinking, and reduce the error rate in the decision-making related to epidemic prevention and control.

Based on the selection of epidemic prevention and control plans, this paper puts forward the following steps:

Step1: Propose feasible solutions.

Epidemic prevention and control policies involve many aspects, each of which is an independent decision. The final promulgated prevention and control policies are the sum of all aspects of decision-making. Among them, the control policies, nucleic acid testing policies, and traffic control programs are the three aspects that should be decided first following the outbreak of an epidemic. After nearly two years of experience, a database of epidemic prevention and control plans has been formed. A set of options suitable for the local epidemic can be selected in the program library, denoted as $X_i (i=1, 2, .., n)$.

Step 2: Select experts and rank the alternatives in preference.

First, identify the experts involved in the decision making of the scheme according to their professional direction (if necessary, experts with similar or less divergent preferences are grouped according to their previous ranking preferences). The expert group is denoted as $E_i(i=1, 2, ... , m)$. Then, based on the feasible alternatives proposed in step 1, each expert makes a comprehensive ranking according to the feasibility, implementation effectiveness, control strength and other aspects of the alternatives.

Step 3: Form expert preference maps (PMs) for the ranking of alternatives.

Based on expert ranking, expert preferences are transformed into PMs according to Definition 1. Define Consensus Evaluation Sequence (CES) and confirm acceptable thresholds for the Group Consensus Index (GCI).

Step 4: Build up a dispute matrix, calculate the pairwise disagreement index (PDisaI) and determine whether the experts reach a consensus.

The dispute matrix is constructed according to the PMs, then the PDisaI can be obtained. If $\mathrm{PDisaI}=0$ or GCI reaches an acceptable threshold, this indicates that experts reach a consensus on the ranking of epidemic prevention and control schemes, which is solved according to Definition 3; otherwise, move on to step 5.

Step 5: Iterate over the preference ranking of experts for alternatives.

Since experts do not reach a consensus on the order of the epidemic prevention and control plan, choose a $\varDelta _{ij}(i<j \ \text {and}\ \varDelta _{ij}>0)$ in descending order of the dispute index. Through negotiations with experts related to the selected $\varDelta _{ij}$, their preferences can be modified for the order of the alternatives. If the two experts do not agree to modify their rankings, then move to another pair of experts with $\varDelta _{ij}>0 \ \text {and} \ i<j$. If none of these experts are willing to change their rankings, proceed to Step 6; otherwise, after obtaining the corrected preference order obtained by the experts, return to step 3.

Step 6: Build an assignment model to minimize the divergence.

The assignment model is established as follows:

$$\begin{aligned}&\text {min}&f =\sum \limits _i\sum \limits _k x_{ik} S_{ik}&\\&\text {s.t.}&\sum _{i=1}^{n}x_{ik}=1,\quad k=1,2,\dots ,n,\\&\sum _{i=1}^{k}x_{ik}=1,\quad i=1,2,\dots ,n,\\&x_{ik}=0,1,\quad i,k=1,2,\dots ,n. \end{aligned}$$

$S_{ik}$ is obtained by Definition 2, indicating the total consensus gap for experts if $X_i$ is to be ranked at position k. Then solve the assignment model.

Step 7: Analyze the results.

According to the results of the allocation model, the final program ranking is obtained. Then, analyze the consensus results and determine the selected epidemic prevention and control solution.

At present, there is no fixed model of COVID-19 prevention-control policies. Different prevention-control policies in different localities and different methods and times for making decisions, so the effectiveness of prevention and control is also different. Based on this, the method proposed in this article is that epidemic prevention experts select effective alternatives in the database, rank their preferences through a preliminary analysis of the local epidemic, and then calculate the consensus evaluation sequence to minimize the difference and get the final plan ranking. The advantages of this method are: First, preference ranking is faster than scoring, which is convenient for relevant departments to respond quickly to emergencies. In addition, this method adds a step to the iterative process of experts’ preferences, that is, the process of negotiating and modifying the preferences of the most divergent schemes and experts to avoid decision-making errors caused by personal factors. Therefore, this method is very suitable for decision-making in the ever-changing new crown epidemic.

## The realization of group decision-making for NAT solutions in epidemic prevention and control

In view of the new situation of the novel coronavirus epidemic, where the mutant strains suddenly spread in a city or area, the epidemic prevention and control center is expected to make decisions quickly.

Next, the selection of a nucleic acid testing scheme is taken as an example to introduce the application of the group decision-making method in the formulation of novel coronavirus prevention and control policies.

Step 1: Propose feasible solutions.

In view of the current COVID-19 situation, epidemic prevention and control experts have screened six alternative plans based on the existing database of epidemic prevention and control plans. The above six alternatives are denoted as $\{X_1, X_2, X_3, X_4, X_5, X_6\}$.

$X_1$: Organize citizens to carry out six rounds of nucleic acid testing on the 1st, 4th, 7th, 10th, 13th and 16th days after the closure of the area. In the nucleic acid sampling, nucleic acid testing in medium and high-risk areas and the containment area will be performed through a one-to-one testing method, which will be prioritized. Testing of the key population is conducted by mixing five individual test samples into one reagent, whilst testing of the common population is carried out by mixing ten individual test samples into one reagent. All work is done off-peak.

$X_2$: Organize a round of nucleic acid detection every three days until there are no new cases or asymptomatic infections. Nucleic acid testing needs to be carried out at least 6 times per person. Single sample testing is adopted in key control areas. The testing of the common population is conducted by mixing 10 individual test samples into one reagent.

$X_3$: Four nucleic acid testings need to be administered for citizens in high-risk areas on the 1st, 4th, 7th and 14th days after the closure of the area. Three NATs (each at least 24 hours apart) need to be conducted for citizens in medium risk areas on the 1st, 4th, 7th and 14th days after the closure of the area. Other people in the city need to be tested once. A single check is used for everyone.

$X_4$: Four one-to-one nucleic acid tests are organized on the 1st, 4th, 7th and 14th days of isolation. The detection objects are confirmed cases, suspected cases, asymptomatic cases, close contacts and resident population, and migrant workers and foreigners within their scope of activities.

$X_5$: Organize citizens in the closed area to carry out NAT five times, on the 1st, 4th, 7th, 10th and 14th days of quarantine. Organize citizens in the control area to carry out NAT four times, on the 1st, 4th, 7th and 14th days of quarantine. Other people in the city must stay at home to quarantine and are not permitted to go out unless necessary.

$X_6$: Organize confirmed cases and close contacts to carry out NAT five times, on the 1st, 4th, 7th, 10th and 14th days of quarantine. Simultaneously, organize for the second close contacts to undergo NAT three times, on the 1st, 4th and 7th days of quarantine. A single check is used for everyone.

Step 2: Select experts and rank the alternatives in preference.

Among the 30 experts in the Center for Epidemic Prevention and Control, select 6 experts who have experience or knowledge related to nucleic acid detection to make decisions on the nucleic acid testing scheme in the outbreak area. The selected six experts are denoted as $\{X_1,X_2,X_3,X_4,X_5,X_6\}$.

The group of experts sorts the six alternatives according to their preferences, and parallel ranking is allowed. The experts’ preferences are as follows:

$$\begin{aligned} \begin{aligned} E_1:X_1\sim X_2\succ X_5\succ X_3\sim X_4\sim X_6, \\ E_2:X_1\succ X_2\succ X_5\succ X_3\succ X_4\sim X_6, \\ E_3:X_1\sim X_3\sim X_4\succ X_2\succ X_5\sim X_6, \\ E_4:X_1\sim X_2\succ X_3\sim X_4\succ X_5\succ X_6, \\ E_5:X_1\sim X_2\succ X_3\sim X_4\sim X_5\succ X_6, \\ E_6:X_1\succ X_2\succ X_3\sim X_5\succ X_4\sim X_6. \end{aligned} \end{aligned}$$

Step 3: Form expert preference maps (PMs) for the ranking of alternatives.

According to Definition 1, the PMs of the experts based on the above experts’ preferences are obtained as follows:

$$\begin{aligned}&\begin{array}{ll}&\begin{array}{l} V^{(1)} \end{array}\\ \begin{array}{l} X_1\\ X_2\\ X_3\\ X_4\\ X_5\\ X_6 \end{array}&{}\left( \begin{array}{l} \{1,2\}\\ \{1,2\}\\ \{4,5,6\}\\ \{4,5,6\}\\ \{3\}\\ \{4,5,6\} \end{array}\right) , \end{array} \begin{array}{l} \begin{array}{l} V^{(2)} \end{array}\\ \left( \begin{array}{l} \{1\}\\ \{2\}\\ \{4\}\\ \{5,6\}\\ \{3\}\\ \{6\} \end{array}\right) , \end{array} \begin{array}{l} \begin{array}{l} V^{(3)} \end{array}\\ \left( \begin{array}{l} \{1,2,3\}\\ \{4\}\\ \{1,2,3\}\\ \{1,2,3\}\\ \{5,6\}\\ \{6,6\} \end{array}\right) , \end{array} \begin{array}{l} \begin{array}{l} V^{(4)} \end{array}\\ \left( \begin{array}{l} \{1,2\}\\ \{1,2\}\\ \{3,4\}\\ \{3,4\}\\ \{5\}\\ \{6\} \end{array}\right) \end{array},\\&\quad \quad \begin{array}{l} \begin{array}{l} V^{(5)} \end{array}\\ \left( \begin{array}{l} \{1,2\}\\ \{1,2\}\\ \{3,4,5\}\\ \{3,4,5\}\\ \{3,4,5\}\\ \{6\} \end{array}\right) , \end{array} \begin{array}{l} \begin{array}{l} V^{(6)} \end{array}\\ \left( \begin{array}{l} \{1\}\\ \{2\}\\ \{3,4\}\\ \{5,6\}\\ \{3,4\}\\ \{5,6\} \end{array}\right) . \end{array} \end{aligned}$$

Identify 1 as the acceptable GCI threshold.

Step 4: Build up a dispute matrix, calculate the pairwise disagreement index (PDisaI) and determine whether the experts reach a consensus.

According to Definition 2, the disagreement matrix $D_0$ is obtained as follows:

$$\begin{aligned} D_0=(\varDelta _{ij})_{6 \times 6}= \begin{pmatrix} 0\quad &{}0\quad &{}6\quad &{}2\quad &{}0\quad &{}0\\ 0\quad &{}0\quad &{}7\quad &{}3\quad &{}0\quad &{}0\\ 6\quad &{}7\quad &{}0\quad &{}2\quad &{}2\quad &{}5\\ 2\quad &{}3\quad &{}2\quad &{}0\quad &{}0\quad &{}2\\ 0\quad &{}0\quad &{}2\quad &{}0\quad &{}0\quad &{}0\\ 0\quad &{}0\quad &{}5\quad &{}2\quad &{}0\quad &{}0\\ \end{pmatrix}. \end{aligned}$$

The pairwise disagreement index (PDisaI) is calculated by formula (2):

$$\begin{aligned} \mathrm{PDisaI}=7\ne 0 \end{aligned}$$

The group consensus index (GCI) is calculated by using formula (1):

$$\begin{aligned} \mathrm{GCI}=\frac{7}{15} \end{aligned}$$

Obviously, the group of experts is not in consensus. The consensus evaluation sequence (CES) at this time is:

$$\begin{aligned} \mathrm{CES}&=[\mathrm{GCI}=\frac{7}{15};\mathrm{MDP}=\{(2,3)\}, \mathrm{PDisaI}=7;\\ \mathrm{MDA}&= \{5\},\mathrm{MDispI}=4] \end{aligned}$$

It shows that a pair of experts with the maximum controversy is $(E_2, E_3)$, whilst the experts express the maximum disagreement on alternative $X_5$.

Step 5: Iterate over the preference ranking of experts for alternatives.

Iteration 1:

As expert 2 and expert 3 have the maximum disagreement, we ask experts 2 and 3 to modify their preferences through negotiation. The preferences changed by them are as follows:

$$\begin{aligned} \begin{aligned} E_2:X_1\succ X_2\succ X_3\sim X_4\succ X_5\sim X_6, \\ E_3:X_1\sim X_2\succ X_3\sim X_4\succ X_5\sim X_6. \end{aligned} \end{aligned}$$

Based on Definition 1, the PMs of the experts are as follows:

$$\begin{aligned}&\begin{array}{ll}&\begin{array}{l} V^{(1)} \end{array}\\ \begin{array}{l} X_1\\ X_2\\ X_3\\ X_4\\ X_5\\ X_6 \end{array}&{}\left( \begin{array}{l} \{1,2\}\\ \{1,2\}\\ \{4,5,6\}\\ \{4,5,6\}\\ \{3\}\\ \{4,5,6\} \end{array}\right) , \end{array} \begin{array}{l} \begin{array}{l} V^{(2)} \end{array}\\ \left( \begin{array}{l} \{1\}\\ \{2\}\\ \{3,4\}\\ \{5,6\}\\ \{3,4\}\\ \{5,6\} \end{array}\right) , \end{array} \begin{array}{l} \begin{array}{l} V^{(3)} \end{array}\\ \left( \begin{array}{l} \{1,2\}\\ \{1,2\}\\ \{3,4\}\\ \{3,4\}\\ \{5,6\}\\ \{6,6\} \end{array}\right) , \end{array} \begin{array}{l} \begin{array}{l} V^{(4)} \end{array}\\ \left( \begin{array}{l} \{1,2\}\\ \{1,2\}\\ \{3,4\}\\ \{3,4\}\\ \{5\}\\ \{6\} \end{array}\right) \end{array},\\&\quad \quad \begin{array}{l} \begin{array}{l} V^{(5)} \end{array}\\ \left( \begin{array}{l} \{1,2\}\\ \{1,2\}\\ \{3,4,5\}\\ \{3,4,5\}\\ \{3,4,5\}\\ \{6\} \end{array}\right) , \end{array} \begin{array}{l} \begin{array}{l} V^{(6)} \end{array}\\ \left( \begin{array}{l} \{1\}\\ \{2\}\\ \{3,4\}\\ \{5,6\}\\ \{3,4\}\\ \{5,6\} \end{array}\right) . \end{array} \end{aligned}$$

Based on Definition 2, the disagreement matrix $D_1$ is built as follows:

$$\begin{aligned} D_1=(\varDelta _{ij})_{6 \times 6}= \begin{pmatrix} 0\quad &{}0\quad &{}2\quad &{}2\quad &{}0\quad &{}0\\ 0\quad &{}0\quad &{}2\quad &{}0\quad &{}0\quad &{}0\\ 2\quad &{}2\quad &{}0\quad &{}0\quad &{}0\quad &{}2\\ 2\quad &{}0\quad &{}0\quad &{}0\quad &{}0\quad &{}2\\ 0\quad &{}0\quad &{}0\quad &{}0\quad &{}0\quad &{}0\\ 0\quad &{}0\quad &{}2\quad &{}2\quad &{}0\quad &{}0 \end{pmatrix}. \end{aligned}$$

PDisaI is calculated using formula (2):

$$\begin{aligned} \mathrm{PDisaI}=2\ne 0 \end{aligned}$$

CGI is calculated using formula (1):

$$\begin{aligned} \mathrm{CGI}=\frac{10}{15}=\frac{1}{3} \end{aligned}$$

Obviously, the group of experts is still not in consensus. The consensus evaluation sequence (CES) at this time is:

$$\begin{aligned} \mathrm{CES}&=[\mathrm{GCI}=\frac{1}{3};\\\mathrm{MDP}&=\{(1,3),(2,3),(1,4),(3,6),(4,6)\}, \mathrm{PDisaI}=2; \\ \mathrm{MDA}&= \{5\},\mathrm{MDispI}=4]. \end{aligned}$$

We can obtain that there are five pairs of experts that have the maximum disagreement: $E_1$ and $E_3$; $E_2$ and $E_3$; $E_1$ and $E_4$; $E_3$ and $E_6$; $E_4$ and $E_6$. Meanwhile, the experts have the maximum disagreement on alternative $X_5$.

Iteration 2:

According to the comprehensive consideration of the parameter values in the above CES, we randomly selected a pair of experts in the six most controversial experts in this iteration, and finally we selected experts 1 and 3 for consultation.

The preferences modified after consultation between experts 1 and 3 are as follows:

$$\begin{aligned} \begin{aligned} E_1:X_1\sim X_2\succ X_5\succ X_3\sim X_4\sim X_6, \\ E_3:X_1\sim X_2\succ X_3\sim X_4\sim X_5\sim X_6. \end{aligned} \end{aligned}$$

According to Definition 1, at this time, the PMs of the experts are as follows:

$$\begin{aligned}&\begin{array}{ll}&\begin{array}{l} V^{(1)} \end{array}\\ \begin{array}{l} X_1\\ X_2\\ X_3\\ X_4\\ X_5\\ X_6 \end{array}&{}\left( \begin{array}{l} \{1,2\}\\ \{1,2\}\\ \{4,5,6\}\\ \{4,5,6\}\\ \{3\}\\ \{4,5,6\} \end{array}\right) \!, \end{array} \begin{array}{l} \begin{array}{l} V^{(2)} \end{array}\\ \left( \begin{array}{l} \{1\}\\ \{2\}\\ \{3,4\}\\ \{5,6\}\\ \{3,4\}\\ \{5,6\} \end{array}\right) \!, \end{array} \begin{array}{l} \begin{array}{l} V^{(3)} \end{array}\\ \left( \begin{array}{l} \{1,2\}\\ \{1,2\}\\ \{3,4,5,6\}\\ \{3,4,5,6\}\\ \{3,4,5,6\}\\ \{3,4,5,6\} \end{array}\right) \!, \end{array} \begin{array}{l} \begin{array}{l} V^{(4)} \end{array}\\ \left( \begin{array}{l} \{1,2\}\\ \{1,2\}\\ \{3,4\}\\ \{3,4\}\\ \{5\}\\ \{6\} \end{array}\right) \end{array},\\&\quad \quad \begin{array}{l} \begin{array}{l} V^{(5)} \end{array}\\ \left( \begin{array}{l} \{1,2\}\\ \{1,2\}\\ \{3,4,5\}\\ \{3,4,5\}\\ \{3,4,5\}\\ \{6\} \end{array}\right) , \end{array} \begin{array}{l} \begin{array}{l} V^{(6)} \end{array}\\ \left( \begin{array}{l} \{1\}\\ \{2\}\\ \{3,4\}\\ \{5,6\}\\ \{3,4\}\\ \{5,6\} \end{array}\right) . \end{array} \end{aligned}$$

According to Definition 2, the disagreement matrix $D_2$ is built as follows:

$$\begin{aligned} D_2=(\varDelta _{ij})_{6 \times 6}= \begin{pmatrix} 0\quad &{}0\quad &{}0\quad &{}2\quad &{}0\quad &{}0\\ 0\quad &{}0\quad &{}0\quad &{}0\quad &{}0\quad &{}0\\ 0\quad &{}0\quad &{}0\quad &{}0\quad &{}0\quad &{}0\\ 2\quad &{}0\quad &{}0\quad &{}0\quad &{}0\quad &{}2\\ 0\quad &{}0\quad &{}0\quad &{}0\quad &{}0\quad &{}0\\ 0\quad &{}0\quad &{}0\quad &{}2\quad &{}0\quad &{}0\\ \end{pmatrix}. \end{aligned}$$

Then, PDisaI is calculated using formula (2) and CGI is calculated using formula (1):

$$\begin{aligned} \mathrm{PDisaI}=2\ne 0, \mathrm{GCI}= \frac{13}{15} \end{aligned}$$

Now, we can obtain that CES is as follows:

$$\begin{aligned} \mathrm{CES}&=[\mathrm{GCI}=\frac{1}{3};\\\mathrm{MDP}&=\{(1,3),(2,3),(1,4),(3,6),(4,6)\}, \mathrm{PDisaI}=2;\\ \mathrm{MDA}&= \{5\};\mathrm{MDispI}=4]. \end{aligned}$$

Based on Iteration 2, there are two pairs of experts that exhibit maximum disagreement. They are experts 1 and 4 and experts 4 and 6. Also, the experts still have the maximum disagreement on alternative $X_5$.

Iteration 3: We ask experts 4 and 6 to modify their preferences through negotiation, but neither of them wants to compromise.

Iteration 4: We ask experts 4 and 6 to modify their preferences by negotiating, but neither of them wants to modify.

Step 6: Build an assignment model to minimize the divergence.

The expert group has not yet reached a consensus on the sequencing of alternatives following the iterative consultations. We minimize differences by constructing an assignment model:

$$\begin{aligned}&\text {min}&f =\sum _{i=1}^{6}\sum _{k=1}^{6}x_{ik} S_{ik}&\\&\text {s.t.}&\sum _{i=1}^{6}x_{ik}=1,\quad k=1,2,3,4,5,6,\\&\sum _{i=1}^{6}x_{ik}=1,\quad i=1,2,3,4,5,6,\\&x_{ik}=0,1,\quad i,k=1,2,3,4,5,6. \end{aligned}$$

Solve the model and the final ranking with minimal disagreement is as follows:

$$\begin{aligned} X_1\succ X_2\succ X_4\succ X_5\succ X_3\succ X_6. \end{aligned}$$

Step 7: Analyze the results.

It shows that the NAT scheme $X_1$ is most suitable for the local epidemic in the face of sudden transmission of mutant strains in the city.

Next, the Cook–Seiford method [5, 6] is applied to solve the decision-making problem of the above epidemic prevention and control, and the results are compared with the results obtained in this paper.

Firstly, the sequential ranking is indicated by the Cook-Seiford Vector (CSV) based on the initial expert ranking of the alternatives.

CSV is represented by medians for parallel rankings, and ultimately assigns an ordinary single number to all experts’ ordering for each alternative. For example, $\{1, 2\}$ is represented as $\{1.5\}$, and $\{1, 2, 3\}$ is represented as $\{2\}$. Thus, the original expert preferences for alternatives in the above examples can be expressed as:

$$\begin{aligned}&\begin{array}{ll}&\begin{array}{l} \mathrm{CSV}^{(1)} \end{array}\\ \begin{array}{l} X_1\\ X_2\\ X_3\\ X_4\\ X_5\\ X_6 \end{array}&{}\left( \begin{array}{l} 1.5\\ 1.5\\ 5\\ 5\\ 3\\ 5 \end{array}\right) , \end{array} \begin{array}{l} \begin{array}{l} \mathrm{CSV}^{(2)} \end{array}\\ \left( \begin{array}{l} 1\\ 2\\ 4\\ 5.5\\ 3\\ 5.5\\ \end{array}\right) , \end{array} \begin{array}{l} \begin{array}{l} \mathrm{CSV}^{(3)} \end{array}\\ \left( \begin{array}{l} 2\\ 4\\ 2\\ 2\\ 5.5\\ 5.5\\ \end{array}\right) , \end{array} \begin{array}{l} \begin{array}{l} \mathrm{CSV}^{(4)} \end{array}\\ \left( \begin{array}{l} 1.5\\ 1.5\\ 3.5\\ 3.5\\ 5\\ 6\\ \end{array}\right) , \end{array} \begin{array}{l} \begin{array}{l} \mathrm{CSV}^{(5)} \end{array}\\ \left( \begin{array}{l} 1.5\\ 1.5\\ 4\\ 4\\ 4\\ 6\\ \end{array}\right) \end{array},\\&\quad \quad \begin{array}{l} \begin{array}{l} \mathrm{CSV}^{(6)} \end{array}\\ \left( \begin{array}{l} 1\\ 2\\ 3.5\\ 5.5\\ 3.5\\ 5.5\\ \end{array}\right) . \end{array} \end{aligned}$$

Secondly, calculate the Cook–Seiford distance between every two CSVs and represent the problem as assigned problem.

We can obtain the distance matrix based on the following formula:

$$\begin{aligned} d_{ik}=\sum _{i=1}^{6}|a^l_i - k| \end{aligned}$$

The distance matrix is obtained as follows:

$$\begin{aligned} (d_{ik})_{6 \times 6}= \begin{pmatrix} 2.5\quad &{}3.5\quad &{}9.5\quad &{}15.5\quad &{}21.5\quad &{}27.5\\ 6.5\quad &{}3.5\quad &{}7.5\quad &{}11.5\quad &{}17.5\quad &{}23.5\\ 16\quad &{}10\quad &{}6\quad &{}4\quad &{}8\quad &{}14\\ 19.5\quad &{}13.5\quad &{}9.5\quad &{}6.5\quad &{}6.5\quad &{}10.5\\ 18\quad &{}12\quad &{}6\quad &{}5\quad &{}7\quad &{}12\\ 27.5\quad &{}21.5\quad &{}15.5\quad &{}9.5\quad &{}3.5\quad &{}2.5 \end{pmatrix}. \end{aligned}$$

Thirdly, minimize the distance among experts:

$$\begin{aligned}&\text {min}&f =\sum _{i=1}^{6}\sum _{k=1}^{6}x_{ik} d_{ik}&\\&\text {s.t.}&\sum _{i=1}^{6}x_{ik}=1,\quad k=1,2,3,4,5,6,\\&\sum _{i=1}^{6}x_{ik}=1,\quad i=1,2,3,4,5,6,\\&x_{ik}=0,1,\quad i,k=1,2,3,4,5,6. \end{aligned}$$

Ultimately, the final consensus ranking is as follows:

$$\begin{aligned} X_1\succ X_2\succ X_5\succ X_3\succ X_4\succ X_6. \end{aligned}$$

Comparison with the results obtained in this paper:

$$\begin{aligned} X_1\succ X_2\succ X_4\succ X_5\succ X_3\succ X_6. \end{aligned}$$

We can obtain two conclusions:

First, it was found that the two methods relating to the ranking position of alternative $X_4$ are controversial. The method presented in this paper moves the location of $X_4$ forward following consultation among the experts. In the case of an outbreak, the order in which NAT should be conducted among the local population is as follows: confirmed cases, suspected cases, asymptomatic cases, close contacts, persons living and active within the scope of the activities of the confirmed cases, persons living and active within the scope of the activities of the close contacts, and all personnel in the region around the affected area. $X_4$ means that nucleic acid screening should be carried out for confirmed patients, close contacts and personnel within their activities at first.$X_5$ and $X_3$ are the nucleic acid screening plans for all people in proximity to the outbreak of the epidemic. Considering the limited human and material resources in the early outbreak, it is necessary and reasonable to give priority to key populations. This demonstrates that the method proposed in this paper is helpful for the prevention and control decision-making.

Second, clearly, Cook–Seiford is a single iterative approach that terminates at the first decision. This approach is equivalent to having only one vote, without providing for iterations of expert preferences. It is unable to identify disputes and then negotiate with controversial experts to minimize disputes, and as such, it cannot guide the expert group to adjust their preferences. Due to the rapidly changing COVID-19 situation and the uncertainty of the spread of the mutant virus at present, the error of using the decision method in this paper is smaller than that of using the existing decision methods in the context of the sudden onset of an epidemic. Therefore, the method proposed in this paper is feasible and more conducive to dealing with uncertain outbreaks.

In summary, the method presented in this paper is more suitable for the decision making of COVID-19 prevention and control solutions.

## Conclusions

Considering the ongoing international COVID-19 situation, this paper uses the preference ranking method in group decision-making to formulate prevention and control programs, minimizing the differences among the expert group to quickly and effectively formulate prevention and control schemes to ensure citizen safety and social stability. On the one hand, the application of this method can optimize the opinions of the epidemic prevention expert group and form a consensus scheme. On the other, it can save crucial time and carry out epidemic prevention and control quickly and effectively. However, there are still some problems in the research conducted in this paper. Firstly, the consensus evaluation sequence index used in this paper still has room for improvement. How to improve the ability to identify differences by improving the consensus evaluation sequence is the next research direction of this article. Secondly, the decision-making method pertaining to epidemic prevention and control proposed in this paper is an empirical decision-making method based on the existing epidemic prevention and control scheme database and experts’ preferences, as well as the current work of this article is prevention and control after the outbreak. How to use the forecasting model and intelligent decision-making to determine the prevention and control solution in the epidemic outbreak area is the direction that required further study and refinement.
